# “Knockout Cancer”: The Impact of Adapted Boxing Training on Quality of Life in Breast Cancer Survivors, a Case Study

**DOI:** 10.3390/jfmk11010071

**Published:** 2026-02-10

**Authors:** Claudia Cerulli, Arianna Murri, Damiano Zizzari, Cristina Rossi, Claudia Maggiore, Stefano Magno, Gianluca Franceschini, Ivan Dimauro, Attilio Parisi, Elisa Grazioli

**Affiliations:** 1 Department of Motor, Human and Health Sciences (DiSMUS), University of Rome “Foro Italico”, Piazza Lauro de Bosis 15, 00135 Roma, Italy; claudia.cerulli@uniroma4.it (C.C.); damianozizzari00@gmail.com (D.Z.); ivan.dimauro@uniroma4.it (I.D.); elisa.grazioli@uniroma4.it (E.G.); 2 Interdisciplinary Department of Wellbeing, Health and Environmental Sustainability—BeSSA Department, La Sapienza University of Rome, 02100 Rieti, Italy; arianna.murri@uniroma1.it; 3 Center for Integrative Oncology, Fondazione Policlinico Universitario A. Gemelli IRCCS, 00136 Rome, Italy; cristina.rossi1@guest.policlinicogemelli.it (C.R.); claudia.maggiore@guest.policlinicogemelli.it (C.M.); stefano.magno@policlinicogemelli.it (S.M.); gianluca.franceschini@policlinicogemelli.it (G.F.)

**Keywords:** exercise oncology, quality of life, boxing complementary therapies

## Abstract

**Background**: Exercise oncology research supports multicomponent interventions as complementary therapies to improve quality of life in breast cancer (BC) survivors. Nonetheless, evidence on sport-specific, engaging approaches, such as boxing-based concurrent training, remains scarce. **Method**: This case study aimed to evaluate the feasibility and safety, and to explore the effects of a 16-week adapted boxing protocol. Two BC survivors with a history of triple-negative BC in treatment were enrolled. The protocol integrated aerobic, strength/power, coordination, balance and boxing-specific exercises through individually adapted, progressive sessions performed twice a week. Outcomes were assessed pre- and post-intervention and included: (I) compliance and adverse event related to the protocol, (II) functional tests (handgrip, single leg stance, 30 s sit-to-stand, trunk/shoulder mobility tests, VO_2_max); (III) body composition parameters (fat mass, fat-free mass,); and (IV) validated questionnaires (EORTC QLQ-C30, FA12, PSQI, BIS, HADS, IPAQ). **Results**: Compliance was high and no serious adverse events were detected. Sit-to-stand performance, as well as VO_2_max and mobility/balance, improved in both patients after the intervention. Participant A showed a favorable body modulation. Participant B, on the other hand, reported a stable weight. Participant A reported large improvements across QLQ-C30 domains, while participant B exhibited mixed results, with improved emotional functioning and pain but declines in cognitive/social functioning. **Conclusions**: The boxing-based concurrent training protocol was feasible, safe, and well-tolerated. Despite the limitation of the case study, the observed functional and psychosocial positive changes highlight the need for adequately larger controlled trials to clarify the training protocol efficacy in order to optimize this exercise approach in BC survivors.

## 1. Introduction

The global burden of cancer incidence and mortality continues to increase, largely due to the aging of the population and the increasing adoption of unhealthy lifestyle behaviors, which furthers cancer risk [1]. Breast cancer (BC) is the most diagnosed cancer and the leading cause of cancer-related death among women globally. In 2020, approximately 2.3 million new cases were reported, representing about one of four new global cancer diagnoses in females [2]. BC is a multifactorial disease influenced by a combination of genetic, hormonal, environmental, and lifestyle-related risk factors [3]. Sedentary behavior is a well-recognized risk factor for BC. In contrast, engaging in regular physical activity has been consistently associated with a reduced risk of developing the malignancy, both in premenopausal and postmenopausal women. The World Health Organization recommends physical activity as a key preventive strategy, noting that, when combined with other lifestyle changes, breast cancer risk could potentially be reduced up to 30% [4]. Epidemiological models suggest that increasing physical activity by even modest levels (e.g., +7.5 MET-h/week) could prevent thousands of cancer cases annually across multiple sites [5]. Moreover, physical activity can reduce the health-related side effect induced by cancer and its treatment, improving patients’ quality of life (QoL) through the entire medical pathway [6,7]. Studies indicate that BC survivors often experience significant challenges across various domains of QoL, necessitating targeted interventions to address these issues [8]. Chronic fatigue is a prevalent issue, with studies indicating that up to 44% of BC patients experience significant tiredness. Localized and general pain are other major concerns, affecting approximately 55% of patients [9]. Moreover, a significant decline is frequently observed across multiple fitness parameters that have a direct impact on quality of life. These include a decrease in maximal oxygen uptake (VO_2_max), which reflects diminished cardiorespiratory endurance and reduced maximal muscular strength and power, especially in the lower and upper limbs, limited joint mobility and range of motion, impaired balance and coordination, and general deconditioning [10]. Such declines in physical fitness may contribute to a loss of independence in daily tasks, higher fall risk, and lower perceived self-efficacy [11]. Moreover, historically, patients undergoing cancer treatment were often advised to rest and avoid physical activity, especially during active therapy phases [12]. However, studies investigating this advice began in the late 80s, with a first randomized controlled study showing physical activity induced improvements in functional capacity, body composition and patient-reported nausea, on BC patients undergoing chemotherapy [13,14]. In the following decades, numerous studies have demonstrated that regular physical activity can mitigate a wide range of cancer- and treatment-related side effects, including fatigue, loss of muscle mass [15], peripheral neuropathy, lymphedema [16], reduced cardiorespiratory fitness [10,17], and psychological distress, helping in the recovery after surgery [18]. This growing knowledge has led major health organizations, such as the American College of Sports Medicine (ACSM) [12,19] and the World Health Organization (WHO), to develop structured, evidence-based exercise guidelines specifically tailored for people living with and beyond cancer. The FITT framework is commonly used to individualize exercise prescriptions in BC rehabilitation. This approach promotes safe and effective physical activity interventions, tailored to the patient’s condition, preferences, and treatment stage. Here we report each parameter with the respective ACSM guidelines [19], personalizing it for BC:

Frequency: For BC survivors, exercise sessions are generally prescribed 3–5 times per week, depending on the individual’s tolerance and baseline fitness level.

Intensity: Moderate intensity training for at least 150 min, or vigorous exercise for 75 min.

Time: Each session timing could differ depending on the protocol frequency and on the fitness status of the patient. According to most published studies, duration usually ranges from 30 to 60 min per session [20].

Type: Combination of aerobic, resistance, and flexibility training. This can be achieved performing specific aerobic, strength and mobility sessions separately, or by combining the different exercise modalities in combined training sessions.

Despite this knowledge, there are still only few BC patients regularly engaged in adapted physical activity protocol. For this reason, there is an urgent need to find a different type of adapted exercise program which could be enjoyable, engaging and feasible for BC patients.

In recent years, unconventional forms of exercise such as contact-based disciplines have attracted growing attention in rehabilitation settings. Among them, boxing-based protocols have shown promising effects in neurological and cardiometabolic patients [21,22]; however, to date, few studies have been conducted on boxing in BC [23]. This case study presents an innovative intervention with the aim of bridging this gap by exploring the feasibility and potential effects of an adapted boxing program for BC survivors.

## 2. Detailed Case Description—Materials and Methods

The primary objective of this case study was to assess the compliance and potential adverse event related to an innovative tailored boxing protocol. Secondly, the intervention aimed to explore whether a structured and supervised boxing-based training program could function as a multidimensional rehabilitation strategy, capable of improving physical and psychological parameters. This study was conducted as pre–post case study without a control group. Two female participants were enrolled according to the following inclusion criteria: women aged between 40 and 65 years, with a confirmed history of BC, currently in the follow-up phase after completing primary treatments (surgery, chemotherapy, and/or radiotherapy). Exclusion criteria included being unable to perform basic activities of daily living, or having cognitive disorders or other disabling comorbidities (e.g., severe heart failure, chronic obstructive pulmonary disease, orthopedic conditions, and neurological disorders). The intervention was conducted between March and June 2025 and received approval from the Research Committee: CAR 223/2024. Both participants provided written informed consent before the initial testing session and the start of the program.

Patients’ Characteristics

Patient A was a 61-year-old woman, employed full time at the time of the intervention. She had been diagnosed in April 2024 with an invasive ductal carcinoma of the left breast, measuring approximately 20 mm on initial imaging (Table 1). The tumor was classified as triple-negative, with an immunohistochemical profile showing ER 2%, PgR < 1%, HER2 0, and a Ki-67 index of 55%. Pathological staging post-surgery was pT2, pN0, with no evidence of lymph node involvement or distant metastases. She underwent a quadrantectomy with sentinel lymph node biopsy, which returned negative for malignancy. Systemic chemotherapy followed, using a dose-dense sequential protocol: four biweekly cycles of Epirubicin (90 mg/m^2^) and Cyclophosphamide (600 mg/m^2^), followed by four biweekly cycles of Paclitaxel (175 mg/m^2^). Radiotherapy commenced and concluded in December 2024. No hormonal therapy was indicated due to the tumor’s receptor-negative profile. From a medical perspective, she had a history of hypertension, managed with low-dose beta-blockers and antihypertensive medication. Her clinical history also reported sinus tachycardia and dyslipidaemia. At the time of enrolment, she was menopausal and not engaged in any structured physical rehabilitation. She did, however, experience discomfort in the operated arm, partly due to the presence of a venous access device (Port-a-Cath), which was still in place during the early stages of the intervention. Regarding her physical activity background, she reported limited involvement prior to diagnosis, with only occasional treadmill walking. Following treatment, she engaged solely in the Knockout Cancer protocol, occasionally performing light mobility exercises at home during weekends. Psychologically, she approached the intervention with caution and low confidence in her physical abilities, though she showed strong motivation to improve. While she had practiced dance in her youth, physical activity had not recently been a consistent source of well-being. Her decision to participate stemmed from a desire to regain physical control and to experience a novel, stimulating rehabilitative approach through boxing.

Patient B was a 54-year-old woman, also employed full time. She had been diagnosed in July 2021 with an invasive ductal carcinoma of the right breast, classified as cT2cN0M0 and presenting a triple-negative molecular profile (ER-/PR-/HER2-), with a Ki-67 index of 40% (Table 1). The diagnosis followed a mammographic detection of a suspicious lesion. She underwent breast-conserving surgery followed by adjuvant chemotherapy and radiotherapy (total dose: 45 Gy). Specific details of the treatment timeline were not available but presumed to align with standard protocols for triple-negative BC. At the time of participation, she was in the follow-up phase and under routine oncological surveillance. The patient also reported a family history of multiple malignancies, although no specific genetic mutations had been identified. Her comorbidities included long-standing autoimmune thyroiditis (Hashimoto’s disease), for which she was treated with levothyroxine sodium (TicheR 75 mcg/day). This therapy was discontinued in September 2024 per endocrinological recommendation and was temporally associated with a notable weight gain of approximately 5 kg. She was not taking any antidepressant or anxiolytic medications, and her cardiovascular and metabolic evaluations indicated no contraindications to exercise. Mild musculoskeletal limitations included residual discomfort and limited range of motion in the operated shoulder, likely due to postsurgical scarring and tissue stiffness. Scar tension was occasionally reported, especially during overhead or extensive stretching movements. Functionally, she had maintained an active lifestyle before her cancer diagnosis, having engaged in a wide range of sports. At baseline, she regularly walked long distances several times per week. Despite initial uncertainty about boxing, she demonstrated high motivation and a strong willingness to participate in structured physical activity, which she viewed as both a health-enhancing and psychologically fulfilling experience.

Based on the prior literature, it was hypothesized that the boxing-based intervention would result in positive outcomes across both physical and psychological domains [24]. In fact, through specific techniques, this sport can improve emotional control, teaching people to control their emotions and not get carried away by anger, as well as self-control and the ability to remain calm even in chaotic situations, teaching them to discern real threats from perceived ones. Both of these characteristics can be very helpful throughout the therapeutic process. Furthermore, it was expected that participants would demonstrate high levels of attendance and engagement, thanks to the dynamic, novel, and stimulating nature of the activity.

### 2.1. Compliance Assessment

The compliance was analyzed through the number of attendances of each participant at the supervised lessons provided for the protocol. Compliance will be expressed as a percentage of the overall lessons. The possible drop-out of the participants and the timing of this drop-out was also recorded, as well as the adverse event related to the protocol.

### 2.2. Testing Procedures

All assessments were administered under standardized conditions and followed a consistent order for both participants. The testing protocol included the following components:

Cardiorespiratory fitness: measured using the 5 min Astrand–Rhyming Step Test [25]. This is an indirect test of maximal oxygen consumption (VO_2_max), through heart rate monitoring. The participant is instructed to step up and down on a 33 cm bench (for women) at a cadence of 90 bpm (dictated by a metronome) for 5 min; each minute and 3 min after the end of the test, the heart rate is recorded.

The test may be stopped before the full 5 min in the event of exhaustion, meaning the participant is unable to maintain the required stepping rhythm for 15 consecutive seconds.

Upper body strength: evaluated through the Handgrip Test (Jamar Plus^®^, Patterson Medical Ltd., Sutton-in-Ashfield, UK), which is representative of the general strength of the subject, and measures the strength of the handheld grip by tightening the dynamometer as tightly as possible. Three attempts for both hands were recorded, and the median value was evaluated [26].

Lower body muscular endurance: assessed via the 30-Second Sit-to-Stand Test; the number of times that the patient could properly get up from a chair and sit back down in 30 s was counted [27].

Balance: evaluated with the Single Leg Stance Test for both legs; patients had to hold the single stance position for 30 s [28].

Flexibility: assessed through the Sit-and-Reach Test [29], Back Scratch Test [30], and Bilateral Trunk Rotation Test.

Anthropometric measurements: body height and weight.

Circumference measurements: waist, hip, measured at standard anatomical landmarks.

Body composition: assessed using bioelectrical impedance analysis (BIA Handy 3000 by DS Medigroup S.r.l. Milano, Italy), with frequencies of 50 kHz and 100 kHz, and the measurement of the following parameters is given: TBW (Total Body Water), ICW (Intracellular Water), ECW (Extracellular Water), FFM (Fat-Free Mass), BMR (Basal Metabolic Rate), BMC (Bone Mineral Content), and PA (Phase Angle).

In addition to physical testing, a battery of validated psychological questionnaires was administered:

EORTC QLQ-C30: the most frequently used cancer-specific health-related questionnaire. It is organized into five different functional subscales (PF: physical functioning; RF: role functioning; EF: emotional functioning; CF: cognitive functioning; and SF: social functioning) and eight symptom items [31];

EORTC QLQ-FA12: evaluates cancer-related fatigue, including physical, emotional, and cognitive dimensions. It consists of 12 items, with four response categories for each item, coded with values from 1 to 4 [32];

Pittsburgh Sleep Quality Index (PSQI): measures subjective sleep quality and disturbances over the previous month [33]. Each component score of the PSQI ranges from 0 to 3, with 3 indicating the greatest dysfunction or disturbance;

Body Image Scale (BIS): a 10-item Body-Image Questionnaire to measure cognitive, behavioral, and affective body image distress, often used in cancer care [34];

Hospital Anxiety and Depression Scale (HADS): detects symptoms of anxiety and depression in clinical populations [35];

International Physical Activity Questionnaire (IPAQ): estimates weekly physical activity levels across different intensity domains [36].

### 2.3. Intervention Protocol

The intervention period lasted 16 weeks, from March to July 2025, and included a total of 33 scheduled sessions.

FITT Framework Report

Frequency: The initial design of the protocol envisioned a frequency of three sessions per week. However, the final implementation averaged two sessions per week (see Supplementary Material). This adjustment represented a pragmatic solution, balancing participants’ occupational and personal commitments with the need for consistent program compliance and physiological progression.

Intensity and Progressive Overload: Cardiovascular intensity was regulated using the modified Borg Rating of Perceived Exertion (RPE) scale (6–20) and was progressively increased throughout the intervention. During the early sessions, aerobic drills were performed at moderate-to-high intensities (RPE 6–7). Following observable physiological adaptation and improved technical execution (around sessions 7–8), training progressed toward higher intensities (RPE 8). In the final phase of the program, participants occasionally worked at submaximal levels (RPE 9), guided by their increased physical capacities and increased psychological confidence. Resistance training was performed at moderate intensities (RPE 7–8), roughly aligned with a 10–12 repetition maximum (RM). Each set was structured to fall within this range, maintaining a 3–4 repetition buffer from muscular failure to ensure safety while promoting musculoskeletal adaptation.

Time: Each training session lasted approximately 90 min, ranging from 60 to 105 min. Session length varied depending on factors such as the day’s planned content, intensity, the participant’s physical or emotional condition, and arrival time at the facility. This flexible structure allowed for individual pacing without compromising program consistency or therapeutic aims.

Type: Training sessions incorporated a diverse array of exercise modalities, including aerobic conditioning, resistance exercises (conducted in a weight room), balance and mobility drills, coordination work, cognitive–reactive tasks, and boxing-specific activities (e.g., heavy bag work and mitt drills). This integrative approach aligned with the multidimensional rehabilitation needs of BC survivors, emphasizing both physical reconditioning and engagement through varied, task-oriented practice.

Sessions Structure and Content

Two main session formats were alternated throughout the intervention, each structured to target distinct physical domains while preserving core boxing-specific elements (See Supplementary Material).

1. Strength-Focused Sessions

These sessions began with a 15 min warm-up phase that included dynamic joint mobility drills for both upper and lower limbs. The central component was a 30–40 min strength training phase, carried out in the gym. This segment featured six resistance exercises targeting major muscle groups, such as hip thrusts, leg press, lat pulldown, and pec deck machine. Sport-specific drills, such as banded torso rotations and medicine ball throws, were also included to functionally prepare participants for the boxing segment while reducing injury risk.

2. Endurance-Focused Sessions

The session started with a 10–15 min warm-up. The central phase focused on cardiovascular endurance through interval-based and functional circuit modalities. The boxing-specific segment was carried out on the ring with dedicated one-to-one sessions with the trainer.

For each patient, pre- and post-intervention fitness data were reported, and for each of them, differences were evaluated using the percentage change, considering the baseline data as the reference. The difference between values at T0 and T1 was analyzed using the following formula: ∆% = (T1 − T0/T0) × 100, calculated with Microsoft Excel.

### 2.4. Compliance Results

Both patients completed the intervention with a high level of compliance. Patient A attended 22 out of 26 sessions (85%). Around session 22, she started to express concerns regarding her ability to maintain consistent attendance in the final phase of the program, due to increasing personal commitments and high summer temperatures. In agreement with the research team, it was decided to anticipate the post-intervention assessments to avoid the risk of prolonged inactivity that could confound results. This adjustment allowed for a more accurate evaluation of the adaptations achieved during the intervention. Patient B completed the full protocol, attending 26 out of 32 sessions (79% compliance rate). All absences were related to personal obligations, except for two missed sessions due to a minor episode of lower back discomfort. Although the symptoms emerged two days after session 27, the patient attributed the issue primarily to prolonged standing over subsequent days rather than to the training itself. Consequently, she did not attend sessions 28 and 29 but returned to full participation thereafter. No further adverse event related to the protocol was reported by the patients.

### 2.5. Quality of Life Results

As reported in Table 2, the EORTC QLQ-C30 results revealed distinct trajectories between the two patients, highlighting individual perceptions and experiences throughout the intervention.

Patient A demonstrated a broad and consistent improvement across nearly all functional and symptom scales: Physical functioning increased by 29.89%, and role functioning by 49.99%. In addition, emotional functioning rose from 50 to 83.3, with even greater gains in cognitive functioning (from 33.33 to 100) and social functioning (from 83.3 to 100). Lastly, global health status improved by 42.88% after the protocol, reflecting an enhanced overall quality of life.

The symptoms scores also improved markedly; results evidenced a decrease in fatigue of 80.04%, in pain of 100% and in insomnia of 66.67%.

Patient B, on the other hand, showed mixed results, with fluctuations across domains. A slight decrease was observed in physical functioning (from 100 to 86.7) and role functioning (from 100 to 83.3). Emotional functioning improved moderately (33.40%), while cognitive and social functioning declined by 33.40% and 20.05%, respectively. Global health status remained unchanged. In terms of symptoms, pain decreased 49.98%, and fatigue remained stable, as well as insomnia, dyspnea, and appetite loss.

**Figure 1:**
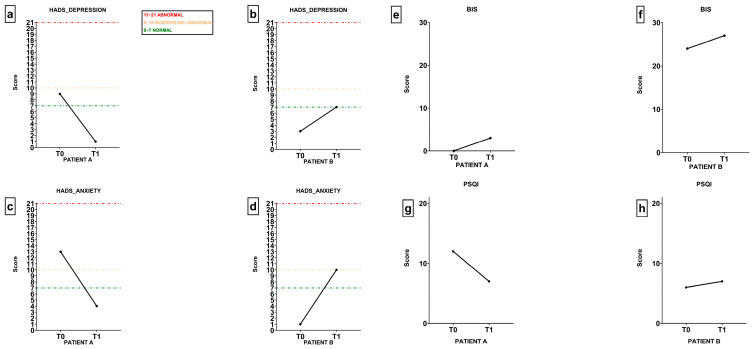
Results of HADS, BIS and PSQI before and after boxing-based protocol: The HADS scoring values are represented through colors: green = normal (score 0–7); yellow = borderline/abnormal (score 0–10); red = abnormal (score 11–21). (**a**) HADS depression score before and after training patient A; (**b**) HADS depression score before and after training patient B; (**c**) HADS anxiety score before and after training patient A; (**d**) HADS anxiety score before and after training patient B; (**e**) BIS patient score before and after training A; (**f**) BIS score before and after training patient B; (**g**) PSQI score before and after training patient A; (**h**) PSQI score before and after training patient B.

According to the results related to fatigue perception (Table 3), patient A showed substantial improvements across all dimensions of the ERTC FA12 questionnaire. Physical fatigue decreased by 71.46%, and emotional fatigue by 100%. Similarly, cognitive fatigue, interference with daily life and social sequelae were erased entirely at the end of the protocol. These changes indicate a pronounced reduction in both the perception and impact of fatigue, supporting the effectiveness of the intervention for this participant. Patient B reported a slight increase in physical fatigue (16.50%). Conversely, she experienced a decrease in emotional fatigue (33.39%) and social sequelae (49.95%). Cognitive fatigue and interference with daily life remained stable. Moreover, patient A’s results related to sleep quality, showing a reduction in PSQI global score from 12 to 7 after the protocol. Patient B showed a stable sleep profile, with the global score slightly increasing from 6 to 7. The Body Image Scale (BIS) results showed a slight increase in the total score after the protocol in both participants, indicating a certain body image concern. Lastly, patient A showed a marked improvement in both subscales of the HADS questionnaire. Her depression score decreased from 9 (classified as borderline/abnormal) to 1 (normal), and her anxiety score dropped from 13 (abnormal) to 4 (normal). Patient B, in contrast, displayed a deterioration in emotional well-being. Her depression score increased from 3 to 7 (just within the normal range), while her anxiety score rose from 1 to 10 after the protocol, transitioning from normal to borderline abnormal (Figure 1).

### 2.6. Functional Results

As reported in Table 4, patient A demonstrated a substantial improvement in aerobic capacity, evaluated through the Astrand–Rhyming Step Test. At baseline (T0), she was unable to complete the full 5 min step test, interrupting the task after 3 min with a peak heart rate (HR) of 138 bpm. At T1, she successfully completed the entire protocol, reaching a peak HR of 126 bpm. Her VO_2_max increased from 20.19 to 22.12 mL/kg/min, reflecting a meaningful enhancement in cardiorespiratory performance. Patient B completed the full test protocol at both T0 and T1, and she slightly increased the VO_2_max from 29.63 to 30.89 mL/kg/min. Both participants completed bilateral handgrip strength assessments at T0 and T1, and both participants showed stable strength in the dominant hand after the protocol (patient A = 0%; patient B = 1.35%), while a notable improvement was observed in the non-dominant hand (patient A = 12.2%; patient B = 9.63%). Even the lower limb endurance and functional capacity, assessed through the 30 s Sit-to-Stand Test, showed improvement after the protocol in both patients (patient A = 60.00% and patient B = 22.22%). Moreover, patient A showed clear improvements in static balance. At T0, she was able to maintain the single leg stance position for 18 s on the left leg; at the end of the intervention, she successfully completed the full 30 s on both legs. Patient B scored the highest score possible before the training and maintained the same score after the protocol. Lastly, patient A demonstrated widespread flexibility improvements. The Sit-and-Reach Test showed an improvement of 16.22%, and the shoulder flexibility improved bilaterally (Back Scratch Test right = −28.12%; Back Scratch Test left = −9.38%), as did the Trunk Rotation Test (right = +54.85%; left = 34.21%). Patient B showed a slight improvement in the Sit-and-Reach Test (0.95%), as well as in the Back Scratch Test (right = 22.50%; left = 22.92%) and the Trunk Rotation Test (right = 41.18%; left = 72.50%).

### 2.7. Body Composition Results

Both patients showed a slight decrease in body weight (patient A: −1.28%; patient B: −0.57%) and BMI (patient A: −1.23%; patient B: −0.38%). A reduction in waist (−6.25%) and hip circumference (−0.50%), as well as in waist-to-hip ratio (−6.25%), was evidenced only in patient A, while patient B showed an increase of 18.44% in fat mass, as reported in Table 5.

## 3. Discussion

To our knowledge, this is one of the first case studies exploring and evaluating the feasibility, safety, and acceptability of an adapted boxing training in BC patients. The compliance was moderately high (85% and 79% for the two participants) and fell within the range typically reported in exercise oncology interventions [22]. It is comparable to the highest adherence levels reported in the combined training and in combat-sport-inspired interventions (85%) [37]. Moreover, no adverse event strictly related to the training was evidenced, and this suggests that this type of protocol could be safe and feasible for BC patients.

The analysis of self-reported questionnaires offered valuable insights into the perceived impact of the intervention. Patient A showed a coherent pattern of improvement across almost all domains of the EORTC QLQ-C30, with marked gains in physical, role, emotional, cognitive, and social functioning, and a 43% increase in global health status. These changes mirror the trends observed in the functional evaluations, suggesting that the tangible improvements in physical capacity likely contributed to a better perception of daily functioning and overall quality of life. The important reduction in fatigue (−80%) and complete resolution of dyspnoea and pain reinforce the notion of a multidimensional recovery, which are in line with those showed in the literature after combined training protocols in BC survivors. Patient A experienced near-complete resolution of all fatigue dimensions, which is coherent with both her improved aerobic capacity and the strength of the lower and upper limbs. In fact, improvements in cardiovascular capacity and strength are usually correlated to lower systemic inflammation and fatigue perception [38]. Sleep quality improvements in patient A might also be related to her reduced anxiety, depression and pain resolution, as emotional distress and nociceptive symptoms are among the strongest predictors of poor sleep in cancer survivors [39]. The slight increase in BIS score may reflect a more accurate and honest self-assessment rather than a true deterioration, possibly accompanied by greater body awareness gained through training and exposure to physical changes, which could have heightened her perception of areas for improvement. Indeed, according to the objectification theory of Fredrickson & Roberts (1997), the shift in focus from body functionality to appearance may increase self-observation and self-criticism, leading to a more self-objectified evaluation of one’s body rather than an actual decline in body image [40,41].

In contrast, patient B exhibited a more heterogeneous pattern of results, encompassing both improvements and declines across the psychological and functional assessments. These findings may reflect the psychological impact of the unfavorable body composition changes observed in the BIA analysis, despite stable weight and anthropometric measurer, or may instead suggest that external psychosocial factors influenced her perceived quality of life during the study period. On the symptoms side, some positive outcomes emerged, including a 50% reduction in pain and stable fatigue levels. The EORTC-FA12 results show a modest increase in physical fatigue, which could be related to the high training intensity and the observed loss of lean mass, potentially exacerbating perceived exertion. Interestingly, emotional fatigue improved by one third, and social sequelae were reduced by 50%, suggesting that, despite physical challenges, the participant may have experienced some psychosocial benefit from the structured intervention and group-based support. On the other hand, HADS scores revealed an increase in both anxiety and depression. This psychological deterioration might be partly attributable to endocrine dysregulation following the discontinuation of thyroid therapy, as metabolic changes are well-recognized contributors to emotional distress [41]. Nevertheless, results from functional parameters showed improvements in the strength of the lower and upper body, suggesting a positive impact of adapted boxing training on physical function.

Despite the encouraging findings, several limitations must be acknowledged when interpreting the results of the present study. First, the sample size is extremely small (*n* = 2), precluding any statistical analysis of the observed changes and restricting generalizability. Given the exploratory nature of the study, all observed changes should be interpreted as descriptive results rather than intervention effects. Furthermore, this design makes the results highly sensitive to individual variability. Second, the heterogeneity of the participants’ medical and endocrine status, particularly the discontinuation of thyroid hormone therapy in patient B, likely influenced both body composition and psychological outcomes, potentially confounding the interpretation of training effects. The intervention duration (16 weeks) may also have been insufficient to induce adaptation for parameters such as body composition and estimated VO_2_max. Lastly, the lack of a usual care control group or one involved in a different type of physical activity makes it impossible to have a direct comparison of the results obtained. Future research should address these limitations by including larger, more homogeneous cohorts, allowing for statistical testing and subgroup analyses (e.g., by menopausal status, treatment type, or endocrine profile).

## 4. Practical Implications

This case study reinforces the importance of adopting an ability-based rather than disability-based approach when prescribing exercise to individuals with chronic health conditions, including breast cancer survivors. Focusing on participants’ capabilities allows practitioners to design training programs that are both empowering and functionally meaningful. The adapted boxing protocol used in this study exemplifies how individualized, progressive, and engaging interventions can enhance physical fitness, mobility, and overall functional capacity. Evidence from other populations with disabilities or chronic conditions similarly highlights the benefits of long-term, tailored exercise programs in promoting improvements in motor performance, body composition, and functional outcomes [42,43]. Integrating these principles into exercise oncology practice may support more sustainable participation and contribute to optimizing quality of life in breast cancer survivors.

## 5. Conclusions

In summary, this case study suggests that a structured boxing-based training protocol may be feasible and acceptable as an alternative multicomponent exercise option for BC survivors. Rather than offering definitive conclusions, these findings highlight the need for carefully individualized exercise prescriptions and for integrated, multidisciplinary approaches capable of addressing the multifactorial nature of survivorship. By combining exercise training with medical, nutritional, and psychological support, future interventions may enhance the consistency of outcomes and mitigate the risk of unfavorable adaptations such as loss of lean mass or deterioration in mental well-being.

## Figures and Tables

**Table 1:** Participant characteristics’.

Variable	Participant A	Participant B
Age	61	54
Weight	70.3 kg	69.6 kg
Height	162 cm	163 cm
BMI	26.7	26.2
Breast Cancer Type and Molecular Subtype	Invasive Ductal Carcinoma; Triple Negative	Invasive Ductal Carcinoma; Triple Negative
Tumor Stage at Diagnosis	pT2pN0M0	pT0pN0M0
Treatments Received	Quadrantectomy, 8 cycles of CH (4 Epirubicin + 4 Paclitaxel), 20 sessions RT	Breast-conserving Surgery, CH (not specified), RT
Time Since Diagnosis (at intervention start)	9 months	4 years
Time Since End of Treatment (at intervention start)	Approximately 3 months	Approximately 3 and 1/2 years
Current Medication	No treatment, follow-up phase	No treatment, follow-up phase
Comorbidities	Hypertension, sinus tachycardia and dyslipidemia	Hypothyroidism (previously treated with Letrozole)

Abbreviations: kg = kilograms; cm = centimeters; BMI = Body Mass Index; CH, chemotherapy; RT, radiotherapy.

**Table 2:** Patient A and B body EORTC QLQ-C30 results.

EORTC QLQ-C30	Patient	T0	T1	%
Physical F	A	66.67	86.6	29.89%
	B	100	86.7	−13.30%
Role F	A	66.67	100	49.99%
	B	100	83.3	−16.70%
Emotional F	A	50	83.3	66.60%
	B	50	66.7	33.40%
Cognitive F	A	33.33	100	200.03%
	B	100	66.6	−33.40%
Social F	A	83.3	100	20.05%
	B	83.3	66.6	−20.05%
Global Health	A	58.3	83.3	42.88%
	B	50	50	0.00%
Fatigue	A	55.6	11.1	−80.04%
	B	33.33	33.33	0.00%
Nausea & Vomiting	A	0	0	NA *
	B	0	0	NA
Pain	A	33.33	0	−100.00%
	B	33.33	16.67	−49.98%
Dyspnoea	A	66.67	0	−100.00%
	B	33.33	33.33	0.00%
Insomnia	A	100	33.33	−66.67%
	B	0	33.33	NA
Appetite Loss	A	33.3	0	−100.00%
	B	33.33	33.33	0.00%
Constipation	A	0	0	NA
	B	0	0	NA
Diarrhea	A	33.3	33.3	0.00%
	B	0	0	NA
Financial Difficulties	A	0	0	NA
	B	0	0	NA

Abbreviations: F = Function, * Not Applicable (it was not possible to applicate the formula)

**Table 3:** Patient A and B EORTC QLQ-FA12 results.

EORTC QLQ-FA12	Patient	T0	T1	%
Physical Fatigue	A	46.6	13.3	−71.46%
	B	40	46.6	16.50%
Emotional Fatigue	A	44.4	0	−100.00%
	B	33.33	22.2	−33.39%
Cognitive Fatigue	A	33.3	0	−100.00%
	B	16.66	16.66	−0.00%
IDL	A	66.6	0	−100.00%
	B	33.33	33.33	0.00%
Social Sequelae	A	33.3	0	−100.00%
	B	66.6	33.33	−49.95%

Abbreviations: IDL = interference with daily life.

**Table 4:** Patient A and B functional evaluation results.

Functional Tests	Patient	T0	T1	%
VO_2_max age-corrected (mL/kg/min)	A	20.19	22.12	9.56%
	B	29.63	30.89	4.25%
30-Second Sit-to-Stand (reps)	A	15.0	24.0	60.00%
	B	18.0	22.0	22.22%
HST-R (Kg)	A	29.6	29.6	0%
	B	29.6	30.0	1.35%
HST-L (Kg)	A	24.6	27.6	12.20%
	B	27.0	29.6	9.63%
S&R (cm)	A	37.0	43.0	16.22%
	B	52.5	53.0	0.95%
Back Scratch-R (cm)	A	32.0	23.0	−28.12%
	B	20.0	15.5	−22.50%
Back Scratch-L (cm)	A	32.0	29.0	−9.38%
	B	24.0	18.5	−22.92%
Trunk Rot-R (cm)	A	31.0	48.0	54.84%
	B	51.0	72.0	41.18%
Trunk Rot-L (cm)	A	38.0	51.0	34.21%
	B	40.0	69.0	72.50%
Single Leg-R (sec)	A	30.0	30.0	0%
	B	30.0	30.0	0%
Single Leg-L (sec)	A	18.0	30.0	66.67%
	B	30.0	30.0	0%

Abbreviations: VO_2_max = maximal oxygen consumption; mL = milliliters; kg = kilograms; min = minutes; reps = repetitions; HST-R = handgrip strength test right; HST-L = handgrip strength test left; S&R = sit and reach; Rot = rotation; cm = centimeters; sec = seconds.

**Table 5:** Patients A and B body composition results.

Body Composition	Patient	T0	T1	%
Weight	A	70.3	69.4	−1.28%
	B	69.6	69.2	−0.57%
BMI	A	26.7	26.4	−1.23%
	B	26.2	26.1	−0.38%
Waist Circ (cm)	A	96.0	90.0	−6.25%
	B	81.0	81.0	0.00%
Hip Circ (cm)	A	100.0	99.5	−0.50%
	B	104.0	104.0	0.00%
Waist-to-Hip Ratio	A	0.96	0.90	−6.25%
	B	0.78	0.78	0.00%
FFM (kg)	A	48.4	47.7	−1.45%
	B	52.6	49.2	−6.46%
FM (kg)	A	22.0	21.7	−1.36%
	B	17.0	20.0	17.65%
BCM (kg)	A	24.9	25.8	3.61%
	B	27.1	25.4	−6.27%

Abbreviations: BMI = Body Mass Index; FFM = fat-free mass; FM = fat mass; BMC = body mass cell; cm = centimeters; kg = kilograms.

## Data Availability

Data available on request due to restrictions.

## Supplementary Materials

The following supporting information can be downloaded at: https://www.mdpi.com/article/10.3390/jfmk11010071/s1, Table S1a,b: Session Examples, two standardized sessions.
