# Supplementary material for: “Knockout Cancer”: The Impact of Adapted Boxing Training on Quality of Life in Breast Cancer Survivors, a Case Study
Source: J Funct Morphol Kinesiol. 2026 Feb 10;11(1):71. doi: 10.3390/jfmk11010071 (PMC12922093; doi:10.3390/jfmk11010071)
Supplement: Supplementary file 1 [file jfmk-11-00071-s001.zip › jfmk-4095276-supplementary.pdf]

Supplementary material

Table S1a,b: Session Examples, two standardized sessions (1a= strength-based, 1b= endurance-based) are provided in the following Tables.

WARM UP (15') (each mobility exercises to be repeated 10 times at least)	Treadmill + Upper body Mobility=<br><br>-1' only walking;<br>-neck mobility (neck flexions, extensions and rotations);<br>-arms along the sides; forward and backwards shoulder rotations;<br>-forward arms crossing to chest openings;<br>-straight arms lateral rotations (extrar.);<br>-"self-hugs" to chest openings;<br>- straight arms lateral rotations (intrar.);<br>-forward arm circomductions;<br>- straight arms forward rotations (extrar.);<br>- backwards arm circomductions;<br>- straight arms forward rotations (intrar.);<br>-arms crossing behind the back;<br>-both arms punch forward;<br>-both arms punch upwards;<br>-both arms punches, 1 forward, 1 upwards;<br>-alternating straight punches forward;<br>-alternating straight punches upwards;<br>-alternating straight punches, 1 forward, 1 upwards;<br><br>-3' Jogging with intensity increase
STRENGHT AND STABILITY (1' 15" rest btwn sets, full rest btwn exercises)	1. STS w/iso' frontal plate hold – 3x30"<br>2. Smith Machine Hip Trust – 3x10<br>3. Isometric SA banded 8kg KB hold + step up and down - 4x15" e/s [15" left, 15" right, rest)

	4. Cable Rear delt openings– 3x12/14<br>5. Pulley w/rope handle – 3x12<br>6. Core: High Plank + 8kg KB drag – 3xMMF
PUNCH SPECIFIC EXERCISES (1’ 30” rest btwn sets, full rest btwn exercises)	1. Lying MB Single Arm Throw – 3x6 e/s<br>2. 5” Iso’ end-punch wall MB straight hold + 2 steps back + MB throw - 3x4 e/s
COMBINATIONS HEAVY BAG (10 each exercise, 5 e/s) *	1. 1-2<br>2. 1-2-slip-2<br>3. 1-2-3<br>4. 1-2-roll-6<br>5. 1-1-4<br>6. Step-in - 3-2 - roll
MITTS ROUND	One 3’ round each

Table S1a: STRENGHT SESSION. Abbreviations: S-T-S= Sit–to–Stand, Iso’ = Isometric; SA= Single-Arm, KB= Kettlebell, MMF= Momentary Muscular Failure; *Combinations were written and often communicated to the patients as numbers, to speed up the 3’ sessions and further stimulate the cognitive involvement. In order, the number were associated to: 1=Jab, 2=Straight Backhand, 3=Lead Hook, 4=Rear Hook, 5=Lead Uppercut, 6=Rear Uppercut, 7= Lead Hook to the body, 8= Rear Hook to the body

WARM UP #1	Walking/Fast Walking+ Upper body Mobility=<br><br>-1’ only walking;<br>-neck mobility (neck flexions, extensions and rotations);<br>-arms along the sides; forward and backwards shoulder rotations;<br>-forward arms crossing to chest openings;<br>-straight arms lateral rotations (extrar.);<br>- “self-hugs” to chest openings;<br>- straight arms lateral rotations (intrar.);

	-forward arm circumduction;<br>- straight arms forward rotations (extrar.);<br>- backwards arm circumduction;<br>- straight arms forward rotations (intrar.);<br>-arms crossing behind the back;<br>-both arms, punch forward;<br>-both arms, punch upwards;<br>-both arms punches, 1 forward, 1 upwards;
WARM UP #2	-Left/Right Galopping (1 gym lap) + Forward/Backward Galopping (1 gym lap)<br>-Knee up and opposite punch throw (1/2 gym lap) + high knees and punches (1/2 gym lap) (x2)<br>-Jogging + 5 jumping jacks when signaled (5x)<br>-Jogging + 5” high knees and punches when signaled (5x)<br>-Jogging with progressive increase of intensity (3 full gym laps)
MIXED CARDIO CIRCUIT (8 stations, 30” in-30” off, 2’ rest) (x2)	1.Basic Jump rope<br>2.Standing Bicep curl + shoulder press (3kg Part.2, 2kg Part. 1)<br>3.step up and down (fast)<br>4.Banded Alternated Forward steps in guard position<br>5.High speed Jump Rope<br>6.Balance on the Tire + slow and controlled shadow boxing<br>7.Airbike Sub-Max intensity<br>8.Farmer walks w/off-balance loaded Trapbar – 3x4 times the length of the gym
COMBINATIONS ON THE HEAVY BAG (10 each, 5 each stance) *	1. 1-2<br>2. 1-2-slip-2<br>3. 1-2-3<br>4. 1-1-2 to the body

	5. 7-3<br>6. 8-3<br>7.. 7-8-3-4<br>8. 3 bouncing in - 2<br>9. 3 bouncing in – 2 to the body<br>10. 1-2 to the body -3
MITTS	One 3' round each on the mitts.

Table S1b: AEROBIC SESSION – *Combinations were written and often communicated to the patients as numbers, to speed up the 3' sessions and further stimulate the cognitive involvement. In order, the number were associated to: 1=Jab, 2=Straight Backhand, 3=Lead Hook, 4=Rear Hook, 5=Lead Uppercut, 6=Rear Uppercut, 7= Lead Hook to the body, 8= Rear Hook to the body
